# Quantification of forest carbon flux and stock uncertainties under climate change and their use in regionally explicit decision making: Case study in Finland

**DOI:** 10.1007/s13280-023-01906-4

**Published:** 2023-08-12

**Authors:** Virpi Junttila, Francesco Minunno, Mikko Peltoniemi, Martin Forsius, Anu Akujärvi, Paavo Ojanen, Annikki Mäkelä

**Affiliations:** 1 Finnish Environment Institute, Latokartanonkaari 11, 00790 Helsinki, Finland; 2 Department of Forest Sciences, University of Helsinki, P.O.Box 27, 00014 Helsinki, Finland; 3 Natural Resources Institute Finland (Luke), Latokartanonkaari 9, 00790 Helsinki, Finland

**Keywords:** Climate change, Forest carbon dynamics, Policy planning, Process-based modelling, Uncertainty quantification

## Abstract

Uncertainties are essential, yet often neglected, information for evaluating the reliability in forest carbon balance projections used in national and regional policy planning. We analysed uncertainties in the forest net biome exchange (NBE) and carbon stocks under multiple management and climate scenarios with a process-based ecosystem model. Sampled forest initial state values, model parameters, harvest levels and global climate models (GCMs) served as inputs in Monte Carlo simulations, which covered forests of the 18 regions of mainland Finland over the period 2015–2050. Under individual scenarios, the results revealed time- and region-dependent variability in the magnitude of uncertainty and mean values of the NBE projections. The main sources of uncertainty varied with time, by region and by the amount of harvested wood. Combinations of uncertainties in the representative concentration pathways scenarios, GCMs, forest initial values and model parameters were the main sources of uncertainty at the beginning, while the harvest scenarios dominated by the end of the simulation period, combined with GCMs and climate scenarios especially in the north. Our regionally explicit uncertainty analysis was found a useful approach to reveal the variability in the regional potentials to reach a policy related, future target level of NBE, which is important information when planning realistic and regionally fair national policy actions.

**Supplementary Information:**

The online version contains supplementary material available at 10.1007/s13280-023-01906-4.

## Introduction

In order to mitigate climate change, many countries and regions are aiming at carbon neutrality by a specific year. In the EU, the target of net zero greenhouse gas emissions has been set to 2050, while Finland, for example, aims to reach carbon neutrality as early as in 2035. Carbon neutrality requires that landscape or nation level greenhouse gas (GHG) emissions are fully compensated with GHG sinks. This goal requires maintenance, or even increase, in GHG sinks. The principal GHG sink in the LULUCF (Land Use, Land Use Change and Forestry) sector results from biological carbon dioxide fixation in forests.

However, yearly fluctuations in weather conditions, forest dynamics (e.g. growth, photosynthesis and disturbances) and industrial demand of wood accumulate as yearly fluctuations in the net biome exchange of CO_2_ (NBE). Forest initial state, harvest levels and environmental conditions also vary spatially. Uncertainty in current forest land NBE estimates and their future projections is, therefore, assumed to be large, yet often neglected. For example, The Official Statistics of Finland reports an uncertainty interval of ($-30\%$, $+ 33\%$), equal to about 10 TgCO_2_eq, in 2020 for the GHG emissions/removals of forest land remaining forest land in Official Statistics of Finland (OSF) (2022). Under large uncertainties, it may be difficult to draw reliable conclusions about the differences between multiple future projections related to, e.g. climate change scenarios and harvest scenarios (Kalliokoski et al. 2018). Uncertainty may also vary depending on the spatial scale (national or regional) and time scale of the projection. The national goals and agreements for future carbon sinks should be based on realistic pathways, also taking into account the risk that the goal is not achieved. The probability of a proposed national pathway to fulfil these goals is determined by the regions where the forests are located. The characteristics of forest productivity, forest use and level of uncertainty in current estimates and in future projections may vary among the regions. Tools and methods to estimate the region level projection uncertainty of carbon balance are, thus, needed to enhance reliable and realistic, as well as regionally fair, policy planning.

Realised state and future dynamics of spatially explicit forest productivity can be modelled with process-based models (PBM). PBMs try to mimic biological processes with simplified equations, and thus, the model parameters are not known exactly, but should have a biological meaning. Model parameters are calibrated with measurements using, e.g. Bayesian models (van Oijen 2017), and the resulting multidimensional, correlated parameter distributions define the parametric uncertainty of the model. Some of the parameters may include more uncertainty than the others. Model parameters may also vary locally, if they are not parameterised according to the location. After calibration of the parameters, PBMs provide a flexible tool to model projections of complex systems under new conditions, such as changing climate, changing management and varying initial and growth conditions, as long as the parameters are calibrated to represent a wide range of conditions comparable to the wide range of expected changes.

The projections should also capture the effect of all sources of uncertainties. Monte Carlo simulations can be used to propagate the uncertainty of inputs and parameters, as well as the variability and uncertainty in weather conditions and harvest levels, through a model to generate time-dependent carbon-balance uncertainty distributions. Uncertainty quantification has been integrated into the carbon budgets, net ecosystem exchange or carbon stock modelling through Monte Carlo simulation, e.g. in Verbeeck et al. (2006), Zhang et al. (2012) and Akujärvi et al. (2019).

In this study, the process-based model PREBAS was used to estimate the NBE and accumulated ecosystem carbon stock projections with uncertainty estimates for all the 18 administrative regions of mainland Finland. PREBAS is designed to model segment and landscape level dynamics of forests. It can be used to simulate the yearly segment level forest management actions under landscape level targets for harvested wood volume by simultaneous simulations of multiple segments. Guidelines for sustainable management (Äijälä et al. 2019) define the possible management actions in individual segments, and the possible harvests are performed until the landscape level target is reached.

The present study is building upon and extending previous work on uncertainty analysis of the PREBAS model. PREBAS combines three sub-models: the PRELES model for photosynthesis, the CROBAS model for structural dynamics and the YASSO model for soil dynamics, which together result the yearly state of the forests. The sub-model parameter distributions have been estimated with Markov Chain Monte Carlo methods, and the resulting model predictions have been validated for boreal forests under different climatic conditions (Liski et al. 2005; Valentine and Mäkelä 2005; Peltoniemi et al. 2015; Minunno et al. 2016, 2019). The main sources of uncertainty in segment level projections of carbon balance were studied in Mäkelä et al. (2020). A schematic flowchart of an individual segment or pixel level simulation with PREBAS is shown in the Supplementary material Fig. S1. In this study, the PREBAS model validated in former studies was used for regional projections with uncertainty quantification.

The objectives of this study were to (1) quantify the uncertainty of projected forest NBE and ecosystem carbon stock in the regions of mainland of Finland; (2) analyse major sources of uncertainty at regional level and with respect to time; and (3) provide an example of the use of uncertainty quantification to estimate probabilities to achieve given targets of forest GHG net sink.

## Materials and methods

### PREBAS model

PREBAS is an open access, process-based model that is suitable for projecting effects of different climatic conditions and harvest scenarios starting from real forest structure data at the landscape level. It combines the PRELES model to estimate forest carbon acquisition through photosynthesis (GPP) and the CROBAS model to allocate GPP to respiration and component growth (Valentine and Mäkelä 2005; Peltoniemi et al. 2015; Minunno et al. 2016, 2019). A flowchart of basic PREBAS simulation for one pixel or segment is shown in Fig. S1. In PREBAS, photosynthesis and evapotranspiration are driven by daily inputs of radiation, temperature, vapour pressure deficit, precipitation and ambient CO_2_ concentration (Peltoniemi et al. 2015; Minunno et al. 2016; Kalliokoski et al. 2018). Output variables are estimated with an annual time step. The model includes tree mortality due to crowding (Reineke 1933) and a random mortality module (Siipilehto et al. 2020) based on forest structure. The ground vegetation biomass depends on site type and below-canopy light availability, which are estimated in a separate ground vegetation model based on empirical results from ground vegetation inventories (Mäkelä et al. 2023). Effects of biotic or abiotic disturbances, or possible nitrogen deficiency, which are likely to affect forest growth negatively, are not included in the current version of the PREBAS model.

Required forest initial state values in PREBAS are pixel or segment level basal area, mean tree breast height diameter, median tree height and proportions of pine, spruce and birch. Also average age, which affects the management decisions, and site type index, which describes site fertility, are required.

Net biome exchange, NBE, is the sum of the net ecosystem exchange (NEE) and the harvested biomass. To calculate NEE, which also includes soil carbon processes, PREBAS has been linked with the soil carbon model YASSO07 through annual litter inputs (Liski et al. 2005). The YASSO model is suitable for mineral soils. The organic soil carbon processes differ from mineral soil carbon processes. However, models suitable for landscape level dynamic modelling of drained organic soil carbon processes, which could be used with existing country level spatial information, do not exist at the moment. The mineral soil carbon processes in YASSO were, thus, replaced with static emission coefficient-based estimates that depend only on the soil type class (drained organic soil), emission type (et) and site type of the location. In the organic soils, the GHG emissions consist of methane (CH_4_), nitrous oxide (N_2_O) and carbon dioxide (CO_2_), see Table 1. Also the soil carbon stock estimated in PREBAS was replaced according to Turunen and Valpola (2020) for forests on organic soils. Lacking a reliable, spatially accurate initial value of the soil carbon stock in drained organic soils, it was assumed to remain constant over the simulation period.

**Table 1:** Drained organic soil emission coefficient mean values and standard deviations by emission type (et). Site types 1–3 include nutrient-rich sites (herb-rich type and blueberry type) and site types > 3 include nutrient poor sites (lingonberry type, dwarf-shrub type and lichen type)

et	Gas	Site type	Unit	$\mu _{et}$	$\sigma _{et}$	References
1	CH_4_	All	gCH$_4$m$^{-2}$year$^{-1}$	0.34	0.12	Ojanen et al. (2010)
2	N_2_O	1–3	gN$_2$Om$^{-2}$year$^{-1}$	0.23	0.04	Minkkinen et al. (2020)
3	N_2_O	$>3$	gN$_2$Om$^{-2}$year$^{-1}$	0.077	0.004	
4	CO_2_	1–3	gCO$_2$m$^{-2}$year$^{-1}$	240	70	Ojanen and Minkkinen (2019)
5	CO_2_	$>3$	gCO$_2$m$^{-2}$year$^{-1}$	− 70	30	

Forest management actions were parameterised on the basis of the best practices for sustainable forest management in Finland (Äijälä et al. 2019; Minunno et al. 2019; Mäkelä et al. 2023). These best practice guidelines describe stand level conditions that trigger a need for thinnings or clearcuts, in terms of species specific mean diameter, dominant height and stand age. In the regional level simulations, the yearly harvests are performed in random order in those segments that meet these conditions, until the region level sum of harvested round wood and energy wood volumes reach the given total region level target volumes. If in any particular region and year, all segments fulfilling the harvest conditions have been harvested before reaching the regional target, no more harvests will be performed. In such a case, the region level target is not reached.

PREBAS model parameters for estimation of forest carbon processes have been estimated with Bayesian methods. The sub-model CROBAS has been calibrated using wide-ranging field measurements including all three main tree species of Finland: Scots pine (*Pinus sylvestris*), Norway spruce (*Picea abies*) and Silver birch (*Betula pendula*) (Minunno et al. 2019). PRELES parameters were calibrated using seven eddy-covariance sites spread across Finland and Sweden (Minunno et al. 2016). YASSO parameters were calibrated using a global database (Liski et al. 2005; Viskari et al. 2021). Under extreme weather conditions, which may occur under severe climate change, the weather variables may occasionally exceed the ranges that were used for the model calibrations. This can lead to erroneous projections.

In this study, the time-dependent model outputs were net ecosystem production and exchange (NEP and NEE, $\hbox {gC m}^{-2}$ $\hbox {year}^{-1}$); nitrous oxide (N_2_O, gN_2_O m^−2^ year$^{-1}$) and methane (CH$_4$, gCH$_4$ m$^{-2}$ year$^{-1}$) emissions from drained organic soils; biomass (kgC ha$^{-1}$) for soil, ground vegetation and trees; and harvested biomass (kgC ha$^{-1}$ year$^{-1}$). The GHG balance of the forests was estimated with net biome exchange (NBE, kgCO_2_eq year $^{-1}$), which is the sum of NEE ($\mathrm{NEE}=-\mathrm{NEP}$), N_2_O, CH$_4$ and harvested biomass (transformed to CO_2_eq year $^{-1}$). Carbon is transformed to CO_2_ by multiplication with 44/12. Nitrous oxide and methane emissions were transformed to CO_2_-equivalent by multiplying with Global Warming potential values 298 for N_2_O and 25 for CH$_4$ (Forster et al. 2007).

### Forest initial values

Spatially explicit initial state variables of the Monte Carlo simulations were based on the Multi-Source National Forest Inventory (MS-NFI) map of the year 2015 (Mäkisara et al. 2019). These data consist of forest structural variables, land class and site type in 16 m $\times$ 16 m pixels. Forests pixels were classified according to the soil type to mineral soil, undrained organic soils and drained organic soils using the National Land Survey data (Haakana et al. 2022). The pixels on undrained organic soils were excluded from the simulations.

To reduce computational effort and to consider realistic, multiple pixel size area management units (similar to management stands), segments consisting of homogeneous pixels were used as computational units in the region level simulations (Haakana et al. 2022). Individual regions consisted of about 420 000–8 694 000 segments, which sum up to about 28 million segments in the country level. The average segment size varied between 0.71 and 0.94 hectares, with the overall smallest segment area 0.026 ha and largest segment area 540 ha (Table 2).

**Fig. 1:**
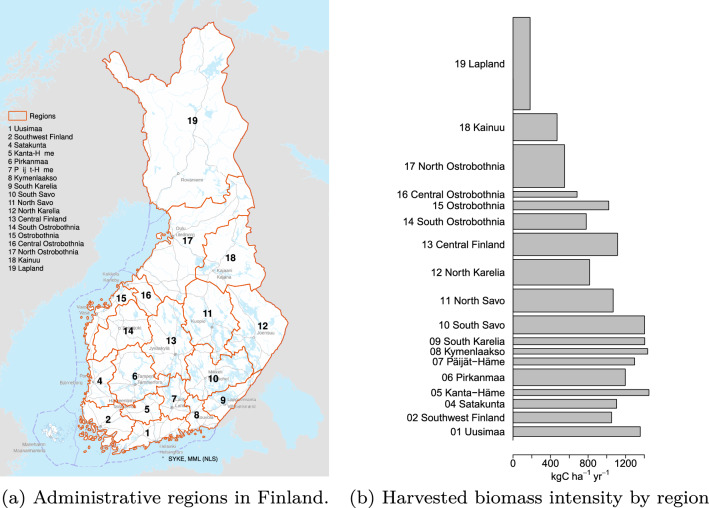
Administrative regions with region IDs in Finland (**a**); Average region level harvested biomass per study area (generated from harvest statistics of period 2015–2021) as bar widths on the *x*-axis and relative region level study area as bar heights on *y*-axis (**b**)

Within the 18 regions, see Fig. 1a, the mean basal area varies between 11 m^2^ ha$^{-1}$ and 18 m^2^ ha$^{-1}$, and mean age varies from 44 years to 91 years (Table 2). Coniferous trees dominate in all the regions, with pine dominating more in the north than in the south, and spruce more in the south than in the north.

**Table 2:** Region level statistics: Study area (segmented forest lands and poorly productive lands), number of segments, mean basal area, mean age and proportion of different species from the total volume

Region		Study area (km^2^)	No. of segments (1000)	mean	Mean age (years)	Volume proportion		
ID	Name			basal area		Pine (%)	Spruce (%)	Birch (%)
				(m$$^2\mathrm{ha}^{-1}$$)				
01	Uusimaa	5761	819	16	51	32	40	18
02	Southwest Finland	6394	864	16	56	46	33	14
04	Satakunta	5294	662	16	51	41	37	15
05	Kanta-Häme	3554	458	17	49	26	49	16
06	Pirkanmaa	10 031	1277	18	51	34	43	16
07	Päijät-Häme	4202	538	18	48	25	49	16
08	Kymenlaakso	3273	413	17	47	41	34	15
09	South Karelia	4147	464	17	44	43	33	15
10	South Savo	10 733	1228	18	49	41	33	17
11	North Savo	13 808	1603	17	50	34	38	18
12	North Karelia	15 335	1642	18	51	46	27	17
13	Central Finland	13 423	1581	18	51	43	33	15
14	South Ostrobothnia	9330	1126	15	54	57	22	14
15	Ostrobothnia	5204	629	16	51	44	30	17
16	Central Ostrobothnia	3442	390	15	58	56	18	17
17	North Ostrobothnia	25 655	3034	14	64	56	18	16
18	Kainuu	16 235	1848	15	62	56	21	15
19	Lapland	55 307	7457	11	93	58	17	13

### Forest management scenarios

The realised harvest levels of Finland were used as the target harvest levels in the simulations for the years 2015–2021, with the total, country level harvested round wood volume varying between 68 and 78 million m^3^ year$^{-1}$ (Natural Resources Institute Finland 2023b). Also the forest chip components that were utilised as energy wood, such as stems smaller than those accepted for round wood, harvest residues and stumps, were removed from the forests. The country level volume of this type of energy wood varied between 7.2 and 10.2 million m^3^ year$^{-1}$ in the period 2015–2021 (Natural Resources Institute Finland 2023a).

For the years 2022–2050, target harvest levels were estimated as average of the previous years in the harvest scenario *BaseHarv*, which describes a case where current harvest level remains the same in the future. Three other harvest scenarios were used to simulate a slightly more intensive harvests (MaxHarv), substantially less intensive harvests (LowHarv) and no harvesting (NoHarv). Target harvest level in scenario *MaxHarv* was set to $1.2 \times$BaseHarv level to describe very intense harvests, in scenario *LowHarv* to $0.6 \times$BaseHarv level to describe moderate harvest levels following the approach described in Huttunen et al. (2022). In scenario *NoHarv* no harvests were performed after year 2021, and even though it describes an unrealistic scenario, it shows the potential carbon fluxes and the uncertainty propagation by region without the effect of harvests, which have been shown in previous studies to be one of the major sources of uncertainty (Mäkelä et al. 2020). Harvests were allocated only to the productive forest land (according to the land class), no fellings were performed in protection areas and poorly productive forest land.

The target harvest level of each scenario was allocated to each region according to the region’s proportion in the actual whole country harvested volume in the years 2015–2021, see Fig. 1b for regional distribution of average harvested biomass. These proportions are based on the assumption that the regional distribution of forest productivity and industrial wood demand remain the same over the simulation period, which may not hold under climate change and other changes.

### Climate scenarios

Three representative concentration pathways, RCP2.6, RCP4.5 and RCP8.5, were used as climate scenarios in the simulations. Under each scenario, five global climate models (GCMs: CanESM2, CNRM, GFDL, HadGEM2 and MIROC) from the fifth phase of the Coupled Model Inter-comparison Project were used in the simulations of climate scenarios (Meehl et al. 2009; Taylor et al. 2012), similar to the settings in Holmberg et al. (2019). The resulting climate scenarios were down-scaled to a $0.2^{\circ }\times 0.1^{\circ }$ longitude–latitude grid and bias corrected using meteorological observations and a quantile-quantile type of bias correction algorithm (Aalto et al. 2013; Räisänen and Räty 2013; Räty et al. 2014).

### Sources of uncertainty

Sources of uncertainty are identified adjusting the uncertainty taxonomy described, e.g. in Kujala et al. (2013) and McGlynn et al. (2022). Uncertainty elements in the simulations were: (1) inputs: (1a) forest initial state and soil fertility and (1b) GCM uncertainty representing structural uncertainty in the used GCMs; and (2) parameters: (2a) model parameters and (2b) harvest target level. The simulation settings are shown in Fig. S2. The input uncertainties varied spatially in each region, while the parametric uncertainties varied only between simulations, i.e. they remained constant over different regions and through simulation time periods. Although the climatic conditions vary spatially, they remain constant under individual GCMs; thus, they were categorised as input uncertainty in this study. The drivers that were set as constant in the simulations were the climate and harvest scenarios, which were categorised as human decision uncertainty. See Table 3 for different types of uncertainty elements.

**Table 3:** Uncertainty elements in the simulation

Type	Source type	Sources	Variation	How do decrease uncertainty
Epistemic	Measurement error or parametric	Initial state sampling	Spatial	More NFI plots, more informative satellite data in MS-NFI modelling. Increase sample size in uncertainty modelling
		Initial age	Spatial	More measurements, partly natural variation
		Site type	Spatial	More measurements, partly natural variation
	Structural	Soil initial state	Spatial	More measurements, partly natural variation
	Parametric	Crown height factor	Simulation	More measurements, partly natural variation
		PRELES	Simulation	More measurements, partly natural variation
		CROBAS	Simulation	More measurements, partly natural variation
		YASSO	Simulation	More measurements, partly natural variation
		Organic soil emission factors	Simulation	More measurements, partly natural variation
		Harvest target level variation	Simulation	More measurements, partly natural variation
Aleatory	Input uncertainty	Climate model type	Driver	Natural variability: not possible to decrease, GCM accuracy: improve climate models
Human decision uncertainty	Uncertain preferences	RCP scenario	Driver	Worldwide climate actions and policy?
		Harvest scenario	Driver	National regulation?

Epistemic uncertainty is due to the lack of knowledge of the system in respect to quantities and processes within the system. Aleatory uncertainty arises because of the unpredictable, random nature of the physical system under study. Human decision uncertainty arises from subjective human preferences and beliefs

#### Initial state

In the spatially explicit, high-resolution direct Monte Carlo simulations, computational effort is heavy even when using the segmented input variables as computation units: The simulations are repeated multiple times in each scenario for each computational unit. With the 28 million segments covering the forest area of this study, the computational effort needed to be reduced.

The MS-NFI pixel level results are outputs of an improved *k* nearest neighbours (i*k*-NN) model, which is based on the NFI field measurements and spatially projected satellite data (Mäkisara et al. 2019). These pixel level variable values can be considered as realisations of the real variable values plus random modelling error. The segment level data used as the initial state variables included also the random segmentation errors and served here as the erroneous initial state variable value population.

In the region level analysis, the focus is on the region level total and average output results, not on the pixel or segment level. For such analysis, each initial value set of the simulations has to be a representative sample of the population of the initial values of that region. To reduce the computational effort in this study, region level simulations were performed for a random set of 20 000 pixels in the region. The pixels were sampled with replacement from the segmented data population using the proportion of the distinct segment areas from the total study area as weights in order to generate a representative sample of the pixels.

Measured error estimates were not available for the mean age of trees in a pixel. Thus, to produce a realistic approximation of the age data precision, a rough estimate for the age uncertainty based on the experience of the authors was used instead. The mean age of trees in the pixel was assumed to be known more precisely for the younger trees than the older trees. For each pixel, they were sampled using a normal distribution with age-dependent standard deviation: $x_\mathrm{age,j} \sim N\left( \mu _\mathrm{age,j},\sigma _\mathrm{age,j}^2 \right)$, where $\mu _\mathrm{age,j}$ is the MS-NFI mean tree age of the pixel *j* and $\sigma _\mathrm{age,j} = 0.1 \mu _\mathrm{age,j}$. Thus, the 95% probability range of 10 years old trees is approximately 8–12 years, while for 100 years old trees it is 80–120 years.

Pixel level site type is also an estimate in the initial state data. Uncertainty in the site type was simulated by re-sampling site type of each sampled pixel at the beginning of each simulation. The model for the site type probability distributions followed the model given in Haakana et al. (2022). The pixel’s site type probability distribution depends on the MS-NFI based site type and sampled mean tree height, mean tree breast height diameter, basal area and proportion of pine trees. The probability distribution was estimated for each pixel according to its structural variable values. The resulting probability distribution was used to sample a new site type for that pixel. The sampled site type was kept constant over the whole simulation period.

The initial values of PREBAS simulations did not include information about the initial state of the soil carbon stock on mineral soils. The soil carbon processes were estimated using the YASSO model combined with the litter outputs from the PREBAS model. In the absence of the measured data of the initial state, it was modelled with assumption of a steady state. Here, the steady state was estimated for each simulation *i* separately, starting from the random initial state variables described above. PREBAS model with randomly ordered repetitions of realised, historical harvest levels and real local weather data from the years 2015–2021 was run until steady state was reached. The steady state, thus, depends on the sampled initial values and model parameters. The same initial state was used for projections of different harvest and climate scenarios.

#### Model parameters

Model parameters include the parameters used in different sub-model components attached in the PREBAS model: CROBAS, PRELES and YASSO. Samples of these parameters have been estimated and validated with measured data in previous studies (Minunno et al. 2016, 2019; Viskari et al. 2021). These samples of parameter values were used as the parameter populations, from which random sample sets for each simulation were re-sampled with replacement for each simulation *i*.

In the PREBAS model, the initial value of the species-dependent volume is a function of the forest structural values given in the MS-NFI data and estimated crown height. Crown height is not included in the MS-NFI data; thus, it has been estimated using empirical equations (Sharma et al. 2017). However, uncertainty of the crown height estimate was not available for this study; thus, a rough estimate for the crown height uncertainty based on the experience of the authors was used instead. Here, the crown height uncertainty was simulated by sampling the crown height factor from normal distribution, $c_{\mathrm {crown\ height}, i}\sim N(1, 0.1^2)$ and using it to multiply the estimated crown height. Here the crown height was assumed to vary between 80 and 120% of the estimated crown height with 95% probability.

The PREBAS and YASSO models are based on assumption of forests growing on mineral soil. However, also forests located on drained organic soils were included in the simulations. The segments located on drained organic soils have been classified according to the site type to correspond with mineral sites of similar fertility, and the organic soil impact on growth was modelled based on this classification. Uncertainty of the classification of mineral and drained organic soils was not simulated in this study.

Uncertainty in the average organic soil emissions was simulated by sampling emission coefficients, $\mathrm{EF}_{\mathrm{et}}\sim N(\mu _{\mathrm{et}}, \sigma _{\mathrm{et}}^2)$, for which the mean and standard deviations for the emission type (et) are given in Table 1. The sampled emission coefficients remained constant during the simulation period and over all sampled pixels and regions of simulation *i*. The soil carbon stock of forests on organic soils was sampled from $x_{\mathrm{soilC, peatland}} \sim N(543 400, 18 500^2)$ [kg C ha$^{-1}$] for all site types (Turunen and Valpola 2020).

#### Climatic conditions

In the climate scenarios RCP2.6, RCP4.5 and RCP8.5, the weather data uncertainty was a result of the variation within different GCM’s: CanESM2, CNRM, GFDL, HadGEM2 and MIROC. In each simulation *i*, one of the five climate models was chosen randomly with equal probability. The same GCM was used over all the regions. This approach allowed computationally efficient validation of the effect of GCM based uncertainty within a limited number of simulations.

#### Amount of harvests

Historical data of the whole country level harvests were estimated based on information given by Natural Resources Institute Finland (2021). The uncertainties in round wood and energy wood statistical harvest levels were simulated by sampling the target level using normal distribution with mean value given in statistics or in harvest scenario specific projected target level, and standard deviation as 2% of the mean (Peltoniemi et al. 2006). The sampled whole country harvest levels were allocated to region level according to the region harvest level proportion in historical data.

#### Notes about uncertainty sources

Separate uncertainty source components given in this section were sampled independent of each other; thus, the possible correlations between different components of uncertainty sources were ignored. In the input data uncertainty, the possible spatial correlation of site type index and age were ignored, as the homogeneous segments were considered spatially independent. Also, the PREBAS model relies on the assumption of no interaction between segments. Classification of forest land class (forest land and poorly productive forest land) in MS-NFI data and classification of forests location to mineral soil or to different types of organic soils were based on high resolution spatial data and were assumed accurate in this study.

The most significant uncertainties that were not included in this study, are forest disturbances such as wind damages, forest fires, snow damages and biotic risks. They are not included in the current PREBAS version, and overall, spatially explicit projections of such events are difficult to obtain.

### Simulation procedure

Forests in the sampled pixels were simulated for time period 2015–2050. Each region was simulated $n_\mathrm{sim} = 300$ times to roughly cover the uncertainty ranges of the given uncertainty elements. To preserve the spatial and time-dependent correlations caused by model parameters, weather and whole country harvest levels, these sampled values were fixed for the $i^\mathrm{th}$ simulation over each region. Spatially distributed initial values were assumed independent between different regions and simulations.

Outputs of the region level simulations consisted of yearly mean values of the pixel level outputs. The whole country level yearly outputs were estimated as a sum of region level output variable values multiplied with corresponding region specific areas. The resulting total output variable values were transformed to per area values by dividing with the forest area of the country.

The sources of variability in the outputs were evaluated using canonical correlation analysis (CCA) that identifies linear relationships between variables (Hotelling and Pabst 1936). The redundancy index (Rd$_\mathrm{ind}$), which takes into account both variance and correlation, was used to express the amount of variance in the output variables that is explained by variance in the input features (Stewart and Love 1968; Weiss 1972; van den Wollenberg 1977). Redundancy index varies between zero and one, and a higher value indicates that the feature explains more the output variable variability. However, no exact interpretation for the index value exists, and thus, the index values were examined only with respect to other index values. Here, the input features were: average volume and age at the beginning of the simulation period (vol0 and age0); climate scenarios (RCP); harvest scenarios (Harv); global climate model (GCM); the PREBAS (CROBAS and PRELES) and YASSO model parameter sets (pCrob, pPrel, pYas), organic soil emission coefficients for carbon, N_2_O and CH_4_ (pECorg) and harvest level uncertainties (pHarv) of each simulation.

## Results

### Dynamics of uncertainty

The harvested biomass projections are equal for all the harvest scenarios over the period 2015–2021, after which, over the period 2022–2050, they depend on the scenario specific target level. The country level harvested biomass, shown in Fig. S3, becomes lower than the target value in the last years of the simulation period under the two most intense harvest scenarios (BaseHarv and MaxHarv). According to the best practices for sustainable forest management in Finland, harvests are postponed if the current forest structure does not allow fellings. These restrictions take place under MaxHarv and BaseHarv in some regions, while in other regions all the target levels remain sustained, see Figs. S4 and S5. These restrictions can then be seen also in the country level total in Fig. S3.

**Fig. 2:**
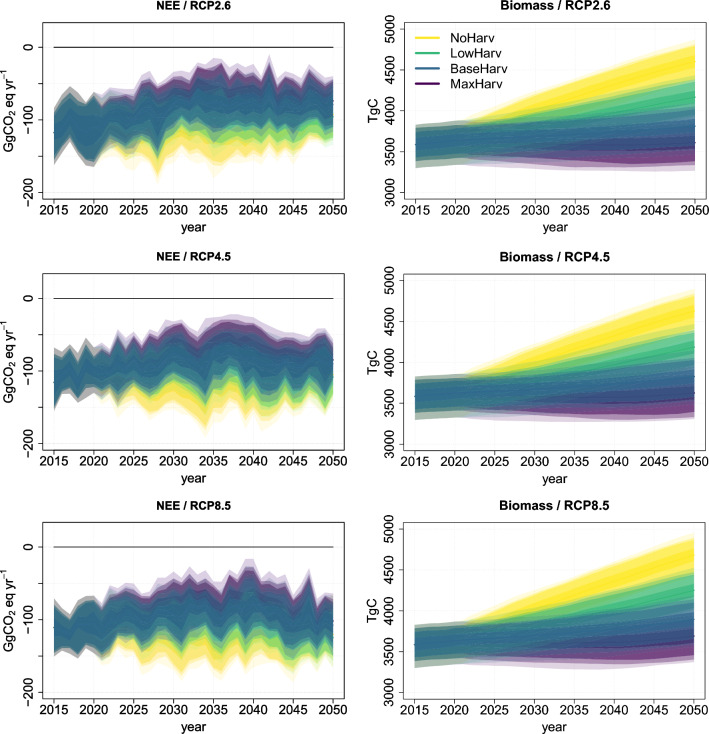
Left column panels: Country level total net ecosystem exchange (NEE); Right column: Country level total ecosystem carbon storage (carbon in trees, ground vegetation and soil). Top row panels: RCP2.6; Middle row: RCP4.5 and Bottom row: RCP8.5. Negative value of the total NEE represents a GHG sink positive a source

At first (years 2015–2021), no impact of the different harvest scenarios are visible in the country level distributions of total net ecosystem exchange (NEE) or total ecosystem carbon stock projections, see Figs. 2 and S6. Over the years 2022–2050, the ranges of total NEE distribution remain the most negative under NoHarv and get larger values under LowHarv, BaseHarv and MaxHarv, from smallest to largest in the same order. Under the climate scenario RCP2.6, the total NEE distribution has slightly increasing trend over time under harvest scenarios BaseHarv and MaxHarv, while under LowHarv and NoHarv the trend stays at a nearly constant level. Under RCP4.5 and RCP8.5, the forest growth has increasing trend under all the harvest scenarios approximately after the years 2030–2035 (not shown here). Also the trend of projected total NEE becomes more negative and a stronger GHG sink. Despite of the small differences among the total NEE trends, the uncertainty in total NEE is large over the whole simulation period under all the climate and harvest scenarios the projected simulated distributions overlap such that no significant differences can be seen between these scenarios.

However, impact of harvest scenarios is visible in the total ecosystem carbon stock, which accumulates as the forest grows. Under scenario NoHarv, the stock becomes larger than under the other harvest scenarios soon after the year 2021, and the more intensive the harvest level is, the smaller the stock remains at the end of the simulation period. The simulated distributions of the stocks under different harvest scenarios overlap over the simulation period for LowHarv, BaseHarv and MaxHarv. However, under NoHarv, the ecosystem carbon stock distribution shows significantly higher values than under other harvest scenarios after the years 2030–2040, depending on the climate scenario.

**Fig. 3:**
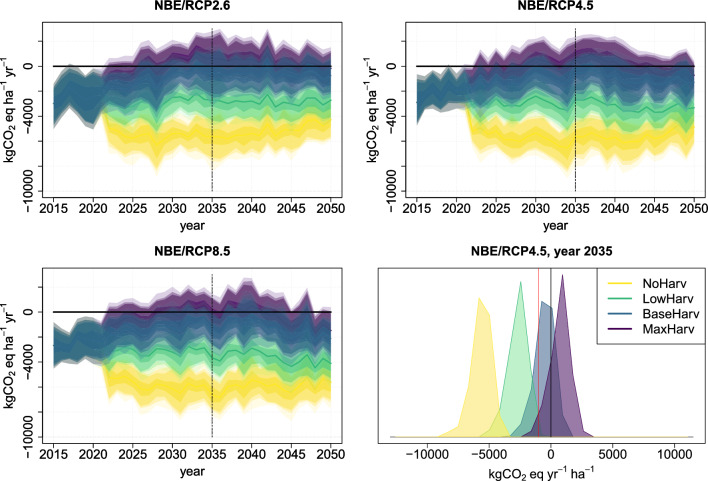
Country level average NBE as a harvest intensity scenario-dependent time-series under RCP2.6 (top left panel); RCP4.5 (top right panel); and RCP8.5 (bottom left panel). In these panels, the vertical line shows the carbon neutrality target year 2035. The year 2035 probability distributions under RCP4.5 are shown in bottom right panel, where red line represents NBE value $-1000$ $\hbox {kgCO}_{{2}}$eq ha$^{-1}$ year$^{-1}$

Although there are no significant differences in projected distributions of NEE between the different climate and harvest scenarios, the empirical distributions of NBE, which is a sum of NEE and harvested biomass, reveal more significant scenario level differences, see Fig. 3. After the year 2021, the simulated distribution of NBE under scenario NoHarv becomes soon substantially more negative than under the other harvest scenarios, independent of the climate scenario. The distributions under LowHarv, BaseHarv and MaxHarv overlap, although the distribution mean is remarkably lower under LowHarv than under BaseHarv and MaxHarv. Similar to NEE projection trends, the NBE distribution ranges are lower, i.e. the net sink is larger, under climate scenarios RCP4.5 and RCP8.5 than under RCP2.6, and under RCP8.5, the NBE projections get the most negative values at the end of the simulation period.

For a specific year, e.g. the year 2035 when Finland aims to be carbon neutral, the country level probability to reach a given carbon net sink target level under different harvest scenarios can be estimated from the year specific empirical distributions shown in Fig. 3 bottom right panel. Here, an example of NBE target level of $-1000$ $\hbox {kgCO}_{{2}}$ eq ha$^{-1}$ year$^{-1}$ is shown as red vertical line. The probability to achieve the target under climate scenario RCP4.5, i.e. forest net sink larger or equal to target sink which means NBE less or equal to the target NBE, is 100% under NoHarv, nearly 100% under Lowharv, 20–30% under BaseHarv, and only a few percent under MaxHarv.

**Fig. 4:**
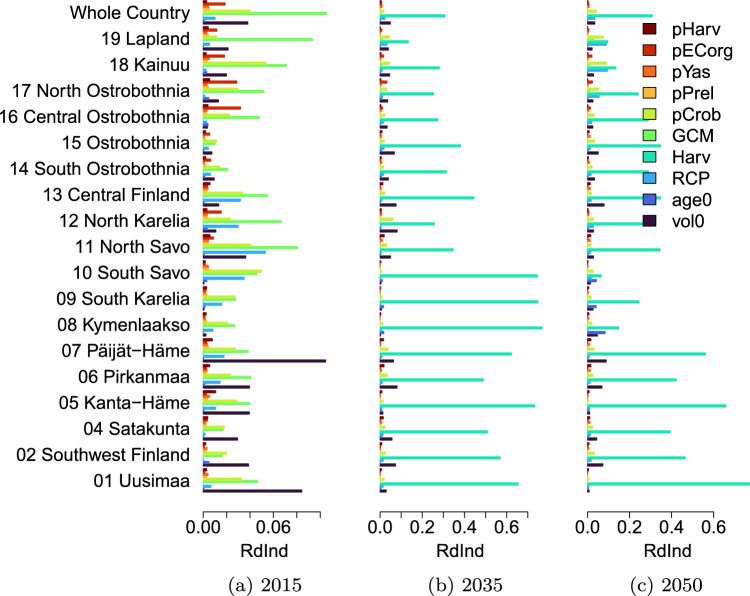
Redundancy index for different sources of uncertainty in NBE in the years 2015, 2035 and 2050. For model parameter sets of CROBAS, PRELES, YASSO, organic soil emission factors and harvest level uncertainty, the highest parameter index value of the set specific Rdind is shown (pCrob, pPrel, pYas, pECorg and pHarv)

### Main sources of uncertainty in NBE

The CCA based redundancy indices, Rd$_\mathrm{ind}$, estimated for country and region level outputs, are shown for NBE in Fig. 4 and for NEE and carbon stocks in the Supplementary material Figs. S7–S10. The unrealistic scenario NoHarv is not included in this analysis, since it would make the dominance of the harvest level uncertainty very high and hide the effect of other uncertainty elements. The dynamics of uncertainty are analysed for three separate time points, years 2015, 2035 and 2050. The region level Rd$_\mathrm{ind}$ values, shown according to the region numbering from 01 to 19 from bottom to top of the figure, follow roughly the south–north gradient of the regions shown in Fig. 1a.

At the beginning of the simulation period, year 2015, the main sources of uncertainty in the whole country level are the uncertainties in the initial volume (vol0), RCP, GCM, CROBAS parameters and organic soil emission coefficients. There is some regional variation in the main uncertainty sources: in the south, the main sources are the uncertainty in initial volume, CROBAS parameters and GCM, while in north, the main sources are the uncertainties in GCM, RCP, and organic soil emission coefficients (mostly in regions with large areas of forests on drained organic soils). In the year 2035, the harvest scenario is the main source of uncertainty for almost all regions, but also uncertainties in RCP, vol0 and CROBAS parameters have substantial effect in some regions. At the end of the simulation period there is more variability in the uncertainty patterns. In most of the regions, the uncertainty in harvest scenario is the main source of uncertainty. In the south-eastern regions 08, 09 and 10, and in the northern regions 18 and 19, the effect of the uncertainty in harvest scenario is less distinct, and also the uncertainty in input features vol0 and age0, and CROBAS parameters have large effect on the NBE uncertainty depending on the region. The regions 08, 09 and 10 are among the ones for which the historical harvest intensities are the largest, see Fig. 1b, and where the harvest target level is not reached under the most intense harvest scenarios, see for such an example Fig. S4. On the other hand, the historical harvest intensities in regions 18 and 19 are the lowest among all the regions, and the different harvest scenarios have the smallest effects on NBE values there.

**Fig. 5:**
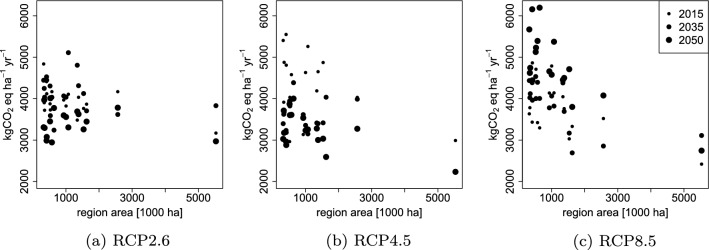
95% range of average Net Biome Emissions in different regions: under **a** RCP2.6, **b** RCP4.5, and **c** RCP8.5 with respect to region area. Dot sizes represent, from smallest to largest dot size, the years 2015, 2035 and 2050

The magnitude of NBE uncertainty varies by region and by time. To study how the uncertainty originating in inputs, model parameters and sampling depends on the region area without the effect of harvests, the 95% range width of the empirical distributions under NoHarv are shown with respect to the region forest area in Fig. 5. At the beginning of the simulation period, the magnitude of the uncertainty depends on the region area, such that the widest uncertainty ranges appear among the regions with the smallest areas. The magnitude of uncertainty remains approximately at the same level under climate scenarios RCP2.6 through the simulation period. Under RCP4.5, the uncertainty range is higher at the beginning of the period, but decreases by the end of the period, while Under RCP8.5, the uncertainty range increases by the end of the period.

### Region level probability distributions

**Fig. 6:**
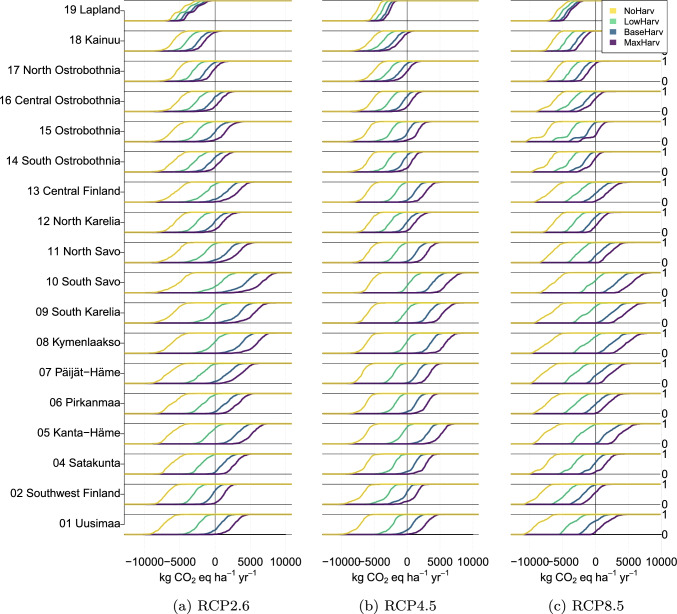
Region level cumulative distribution function of the probability to achieve a NBE value in the *x*-axis under different climate and harvest scenarios

Region level probability distributions are shown as empirical cumulative distribution functions (eCDF) for the year 2035 in Fig. 6. The figure shows the probability (from zero to one) of the NBE to be less than the value on the *x*-axis under different harvest scenarios. For instance, the eCDF curve shows that there is approximately a 100% certainty that in Region 01 (Uusimaa, the bottom row panels), NBE is less than $5000\ \mathrm{kgCO}_2 \mathrm{eq\ ha}^{-1}\ \mathrm{year}^{-1}$ under harvest scenario NoHarv and climate senario RCP2.6 and less than $7000\ \mathrm{kgCO}_2 \mathrm{eq\ ha}^{-1}\ \mathrm{year}^{-1}$ under harvest scenario NoHarv and climate scenario RCP8.5 in the year 2035. Under harvest scenario BaseHarv and climate scenario RCP2.6, Region 01 is a net source with nearly 100% certainty, while under harvest scenario BaseHarv and climate scenario RCP8.5, it is a net sink with approximately 50% certainty. Similarly, for each region, the probability to reach a given NBE target level depends on the climate and harvest scenarios.

The effects of climatic conditions on forest growth can be seen in the NBE (equal to NEE) under harvest scenario NoHarv results in Fig. 6. The potential for the strongest GHG sinks is in the southern regions, which are also the regions with the highest harvest levels related to region forest area (Fig. 1b). Under the harvest scenario MaxHarv, the estimated NBE is positive, meaning a GHG source, in the most southern regions.

The changing climate increases the estimated forest productivity, which increases the probabilities to reach lower NBE values under all the harvest scenarios. However, in the most intensively harvested regions, only harvest scenarios NoHarv and LowHarv result in high probabilities of negative NBE levels, independent of the climate scenario.

## Discussion

This study shows that one of the major sources of uncertainty in NBE in the beginning of the simulation period is the uncertainty in the GCM, which affects in practise the net ecosystem exchange NEE. The climate scenarios start to play a more significant role only around the mid-century. The substantial differences between NBE projections result mainly from the level and allocation of future harvests. NBE was estimated to $-51.0$ TgCO_2_eq $\pm 50.3$ TgCO$_2$eq in the year 2020 under the climate scenario RCP2.6, under RCP4.5 to $-42.3$ TgCO$_2$eq $\pm 21.8$ TgCO$_2$eq, and under RCP8.5 to $-47.2$ TgCO$_2$eq $\pm 37.4$ TgCO$_2$eq. NBE of the year 2020 was also estimated using the real weather conditions between the years 2015–2021 using similar Monte Carlo simulations as described in this study, which resulted to NBE equal to $-$ 36.6 TgCO$_2$eq $\pm 32$ TgCO$_2$eq. These uncertainty intervals are substantially higher than the approximate 2020 estimate of $\pm 10$ TgCO$_2$eq cited above (Official Statistics of Finland (OSF) 2022).

The dynamics of uncertainty vary by region, depending on the harvest intensity in relation to forest structure, region area, and with respect of the projected time. Overall, at the beginning of the simulation period, the uncertainty in the initial forest structure, GCMs, and the CROBAS model parameters have a large effect, and larger ranges of uncertainty appear in smaller regions than in larger ones. By the mid-century, the uncertainty in harvest scenarios play the most important role, while also uncertainty in climate scenarios, initial forest structure and CROBAS parameters play an important role in some regions. The uncertainty in climate scenarios affect only the northern regions by the mid-century.

This study shows that the epistemic uncertainty elements, for which the uncertainty can be at least partly decreased using more measurements and improvement of the knowledge about the model structure (see Table 3), have generally a major role only at the beginning of the simulation period. The uncertainty in projections until the mid-century is mainly caused by aleatory natural variation and human decision uncertainties, which are difficult to decrease. However, the main types of uncertainty elements vary among the regions.

This study shows similar patterns for uncertainties as previous case studies in Finland. For instance, the uncertainty related to gross primary production (GPP), which is the source of all carbon in forest ecosystems, and heterotrophic respiration, have been estimated under different GCMs, emissions and forcing scenarios (both RCP and SRES) in Kalliokoski et al. (2018). In their study, GCM variability was the major source of GPP prediction uncertainty until 2060, only after which the emission pathway became the dominant factor. Similarly, uncertainties in GPP projections were substantially affected by the climate models, climate scenarios and management actions, while the ecosystem model parameters played a smaller role in Mäkelä et al. (2020). Also Vauhkonen and Packalen (2018) pointed out that the level and allocation of the future harvest cause considerable variation in the carbon stored and extracted. The management influences the projection uncertainty more in Southern Finland than in Northern Finland (Mäkelä et al. 2020). They showed that the impact of climate models was relatively constant during the whole period until the year 2100, while the importance of climate scenarios increased towards the end of the simulation period. The GPP and respiration trends showed differences between RCP4.5 and RCP8.5 both in the south and in the north starting from the year 2040, but the uncertainty of these differences with yearly variation remained large and overlapping until the end of the century.

In Finland, carbon-balance projections under different harvesting and climate scenarios of six empirical or process-based models have been compared in the Finnish Climate panel report by Kalliokoski et al. (2019). The results showed wide variability between the results of individual models. All the compared models had been calibrated, tested or developed from existing measurements, but there is no knowledge of their accuracy in future projections, where environmental conditions and processes may differ from the current assumptions. Overall, instead of just one model, the future projections used in policy planning should be based on ensembles of models that complement each other in their response to environmental drivers. Similarly, in Mahnken et al. (2022), a multi-model ensemble mean provided more realistic daily productivity responses to environmental drivers than any single individual model.

Current state and productivity of forests, as well as future productivity and harvest levels vary greatly among regions. Some regions close to forest industry have been long under intense harvests, which have greatly changed the forest structure from its natural state. Such regions are located in south and south-east Finland, where the soil type and site type are beneficial for growth. According to the results of this study, these characteristics may also have an effect on main sources of uncertainty. This information is useful when planning to reduce uncertainty in the regional and whole country level projections. It is also important that the projections of forests under different management policies and climate scenarios are down-scaled to region level analysis which integrates the local conditions to regional potentials for forest carbon balance. This approach emphasises and reveals the spatially explicit risk of not meeting given target with the modelled measures, which can, in the long run, also improve general confidence in modelled projections.

This study shows an example of a regionally explicit modelling procedure, which can be used to estimate region and nation level potentials for different proposed pathways to reach the goals of carbon sequestration. A similar approach can be utilised in all countries for which a locally calibrated ecosystem model and spatially explicit initial state data exist. Overall, region level projections of ecosystem processes give a better insight into the characteristics of different regions, and can reveal possible conflicts and links between multiple demands of forests, such as carbon sequestration and timber use in forest industry. Such policy incoherence can render policy targets unfeasible and even threatens the sustainability of forest ecosystems, especially in timber-producing countries (Blattert et al. 2023).

Overall, Monte Carlo simulations are an efficient tool to project the spatially varying outputs of a complex non-linear process-based model under multiple uncertain initial values, parameters and environmental conditions (Raychaudhuri 2008). However, with a large number of spatially distributed segments, the number of simulations in Monte Carlo analysis needs to compromise with the accuracy gained by large sample size (number of simulations and number of simulated segments) and the computational time. In this study, the computational effort of Monte Carlo simulations with the sample set size and number of iterations applied was approximately 80–100 h for all the scenarios per region using parallel computing in a supercomputer.

As shown in Fig. 2, a substantial increase in NEE is predicted in the PREBAS simulations when RCP4.5 and RCP8.5 are assumed. A major issue is, thus, if other factors might limit this growth in the long term and affect overall sustainability of the system. Critical climate-sensitive risks to forest stability, long-term carbon processes and biodiversity include disturbance caused by extreme weather conditions (e.g. fire, drought, strong winds), biotic factors, invasive species, and large-scale demographic shifts (e.g. elevated mortality rates, species turnover and/or physiological limits to growth or regeneration). Such climate related large-scale risks and patterns are currently not well understood, but they are likely to increase under climate change (Anderegg et al. 2022; Venäläinen et al. 2020). Modelling methods, therefore, need to be developed to quantify the frequency with which, e.g. insects, disease and fire interact and how these relationships change in the future (Parker et al. 2006).

Harvesting of biomass permanently removes nutrients from the forest ecosystems. From a sustainability point of view, the removal of nutrients in the scenarios should, therefore, also be compared with the long-term supply. Nitrogen (N) is generally a growth-limiting factor in boreal ecosystems (Hyvönen et al. 2008), and although N mineralisation may increase under a warmer climate (Wright 1998), the long-term supply is uncertain. The effect of the nutrient limitation is currently being incorporated in PREBAS and will be included in the analysis in the near future. Also, different types of stochastic disturbances are being implemented in the model.

Under extreme climate change, the weather inputs may be out of the ranges that were used for calibration of the model parameters. This should be noted especially in the case of the climate scenario RCP8.5, for which the PREBAS model projections may be erroneous. This, combined with the lack of the stochastic disturbance modules in the model, is likely to increase the uncertainty of the projections from those shown in this study and probably turn the forest from sink to source.

Another source of uncertainty likely leading to underestimation of NBE is that the drained organic soil emissions coefficients applied in this study (Table 1) are static. While dynamic emission coefficients are not available, especially CO_2_ emissions are likely to strongly increase along with the warming climate. Warming climate directly enhances decomposition and leads to drying of peatlands, causing emissions to be higher (Hiraishi et al. 2014).

Spatially distributed initial values of the simulations were chosen to be as accurate as possible. However, the MS-NFI data are results of a model with modelling errors and averaging effect, based on field data with measurement errors. The MS-NFI data is based on k-NN model, which tends to under-estimate the number of youngest and oldest forests. Especially the lack of young forests may affect the estimated near future carbon-balance projections (Haakana et al. 2022).

## Conclusions

The Monte Carlo simulations integrating several sources of uncertainty in initial values and model parameters, and variability and uncertainty in environmental conditions give a wide overview of the processes and their uncertainties. They also describe how the uncertainty in our knowledge about the current state of forests and projected climate and management actions affects the uncertainty in future state of forests. The resulting probabilities can be used to estimate the potential to achieve given target levels of carbon budgets. Our study also points out the need to broaden the discussion of LULUCF sector GHG emission levels from a one value per scenario approach to consideration of probabilities and overall risk analysis. Regional level uncertainty analysis gives more insight in the local conditions and potentials for carbon sequestration, including the risks for over-shoots and significance of different uncertainty elements.

Multiple studies have shown that the uncertainty in the net ecosystem exchange estimates is high, and this needs to be acknowledged in the policy planning. Development of a road map to the future target to achieve carbon neutrality should be based on multiple different modelling approaches supplemented with uncertainty estimates, not only on one model with a deterministic point value result. This information can be given as risk assessment of the decision not to meet given targets.

### Supplementary Information

Below is the link to the electronic supplementary material.

Supplementary file1 (PDF 338 kb)

## Data Availability

Forest net biome exchange and carbon stock projections by the regions of mainland Finland, DOI 10.5281/zenodo.7850309
