# Correction to: Quantification of forest carbon flux and stock uncertainties under climate change and their use in regionally explicit decision making: Case study in Finland

**DOI:** 10.1007/s13280-023-01919-z

**Published:** 2023-09-12

**Authors:** Virpi Junttila, Francesco Minunno, Mikko Peltoniemi, Martin Forsius, Anu Akujärvi, Paavo Ojanen, Annikki Mäkelä

**Affiliations:** 1 Finnish Environment Institute, Latokartanonkaari 11, 00790 Helsinki, Finland; 2 Department of Forest Sciences, University of Helsinki, P.O.Box 27, 00014 Helsinki, Finland; 3 Natural Resources Institute Finland (Luke), Latokartanonkaari 9, 00790 Helsinki, Finland

**Correction to: Ambio** 10.1007/s13280-023-01906-4

In the original publication of the article, the three right panel images of Fig. 2 were mistakenly published with the typo error: GgC appeared instead of TgC. The correct version of Fig. 2 is provided in this correction.

**Fig. 2:**
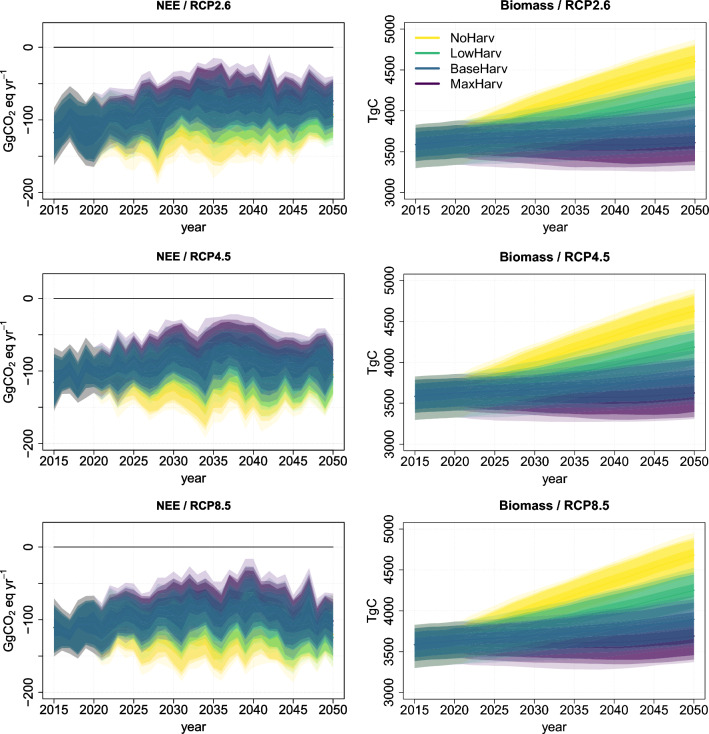
Left column panels: Country level total net ecosystem exchange (NEE); Right column: Country level total ecosystem carbon storage (carbon in trees, ground vegetation and soil). Top row panels: RCP2.6; Middle row: RCP4.5 and Bottom row: RCP8.5. Negative value of the total NEE represents a GHG sink positive a source

The original article has been corrected.

