# Supplementary material for: Quantification of forest carbon flux and stock uncertainties under climate change and their use in regionally explicit decision making: Case study in Finland
Source: Ambio. 2023 Aug 12;52(11):1716–33. doi: 10.1007/s13280-023-01906-4 (PMC10562356; doi:10.1007/s13280-023-01906-4)
Supplement: Supplementary file 1 — Supplementary file1 (PDF 338 kb) [file 13280_2023_1906_MOESM1_ESM.pdf]

Ambio

Supplementary Information

This supplementary information has not been peer reviewed.

Title: **Quantification of forest carbon flux and stock uncertainties under climate change and their use in regionally explicit decision making: Case study in Finland**

Authors: Virpi Junttila, Francesco Minunno, Mikko Peltoniemi, Martin Forsius, Anu Akujärvi, Paavo Ojanen, Annikki Mäkelä

1. FIGURES

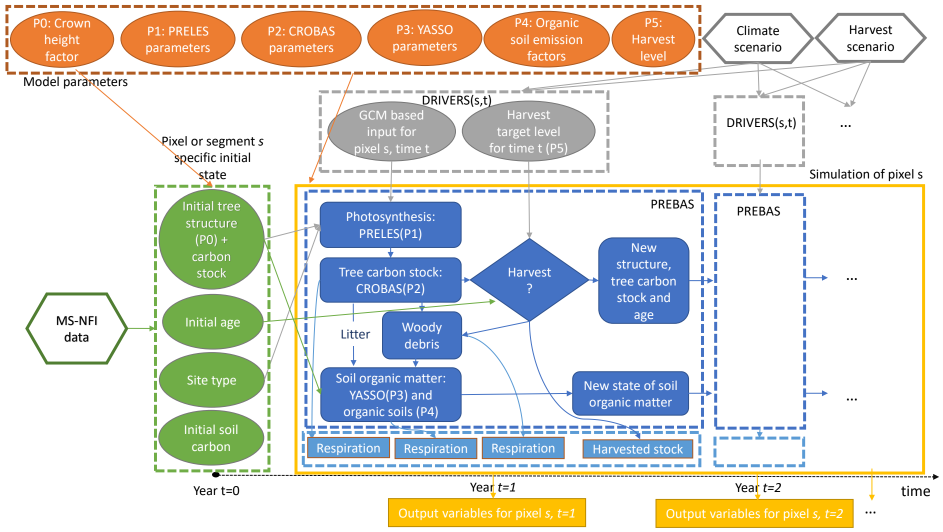

Fig. S1. Flowchart of pixel (or segment) level modelling procedure. The components set by the user are the harvest and climate scenarios, and the source of structural initial state data. The use of different parameters shown in the orange box in the model sub-components is indicated with indices P0 - P5. The spatially varying, pixel or segment specific initial state values are shown with green colour. The yearly drivers are shown in the gray box and the PREBAS model procedure is shown in the blue box. The individual simulation *i* is shown in the yellow box.

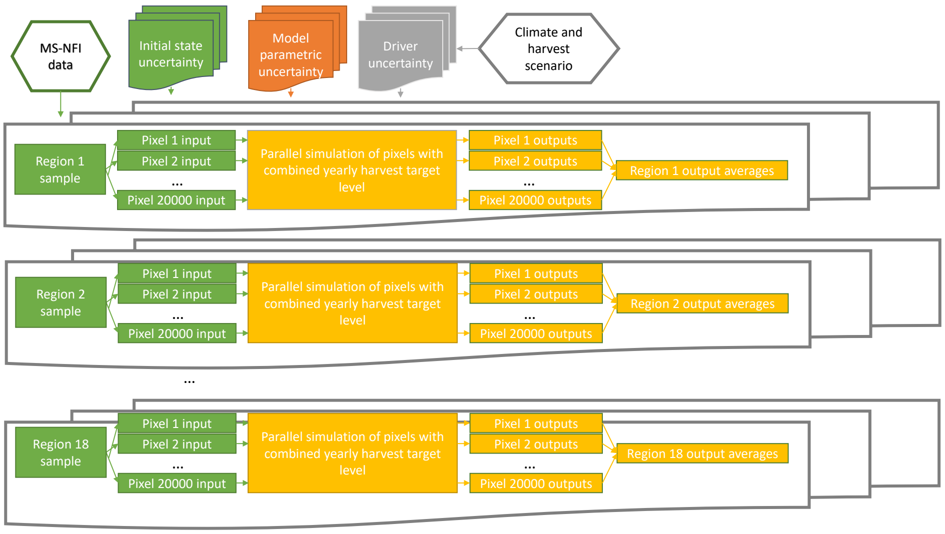

Fig. S2. Flowchart of region level simulation procedure with uncertain inputs, drivers and parameters. The components set by the user are the harvest and climate scenarios, and the source of structural initial state data. The sources of uncertainty for simulation i under individual scenario settings are shown in the top row.

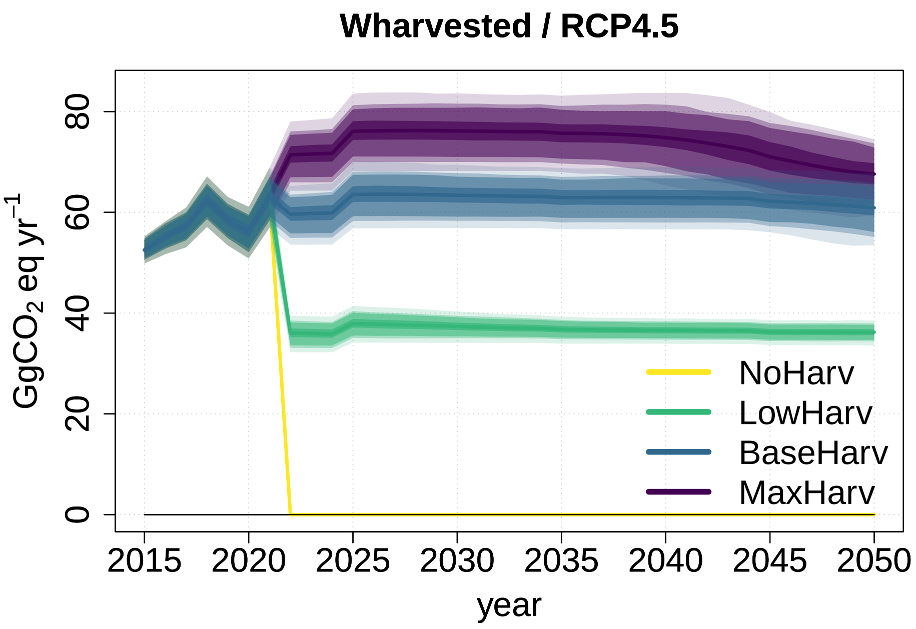

Fig. S3. Country level total harvested biomass ($GgCO_2$eq yr^{-1}) under RCP4.5.

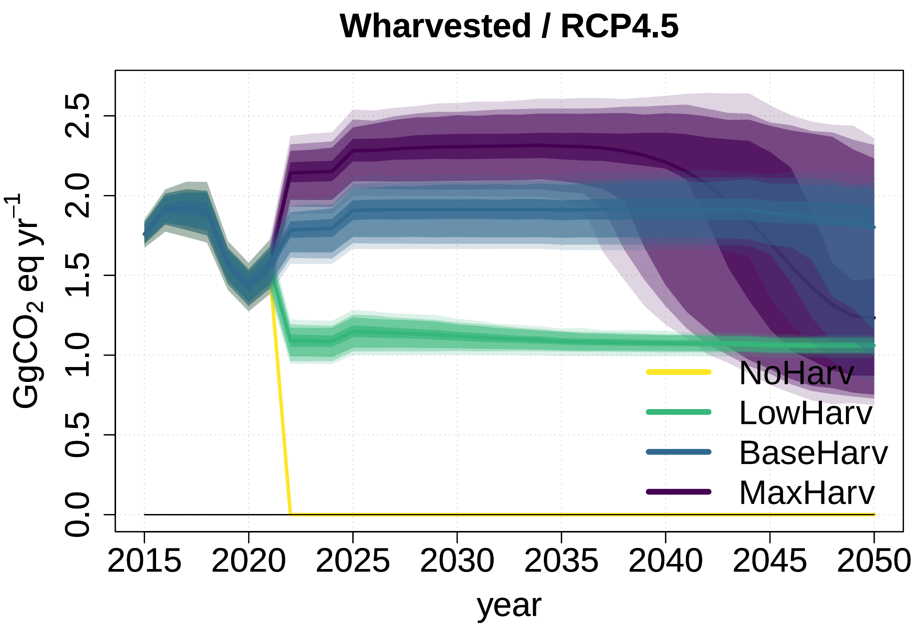

Fig. S4. Kymenlaakso region total harvested biomass ($GgCO_2$eq yr^{-1}) under RCP4.5.

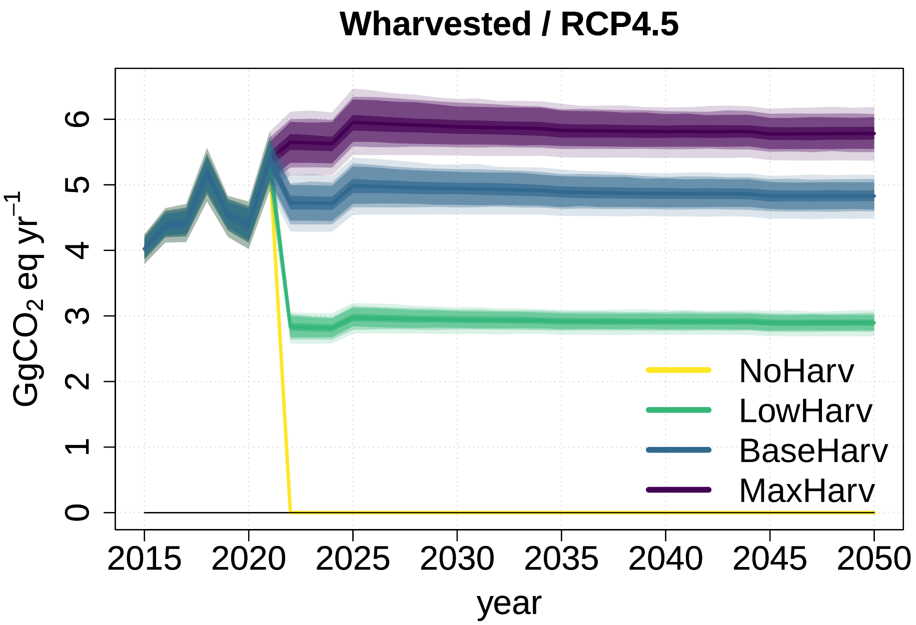

Fig. S5. North Karelia region total harvested biomass ($GgCO_2eq\ yr^{-1}$) under RCP4.5.

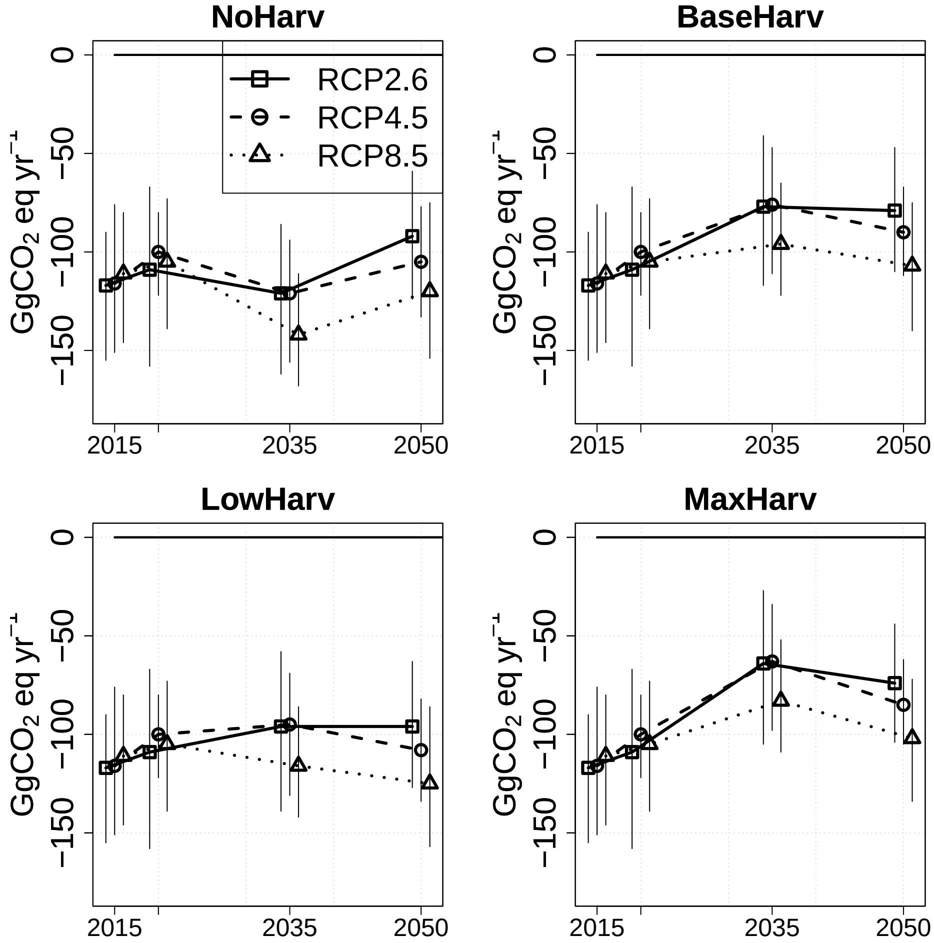

Fig. S6. Harvest scenario specific mean values and 95% uncertainty ranges of total NEE (GgCO_2eq yr^{-1}) under climate scenarios.

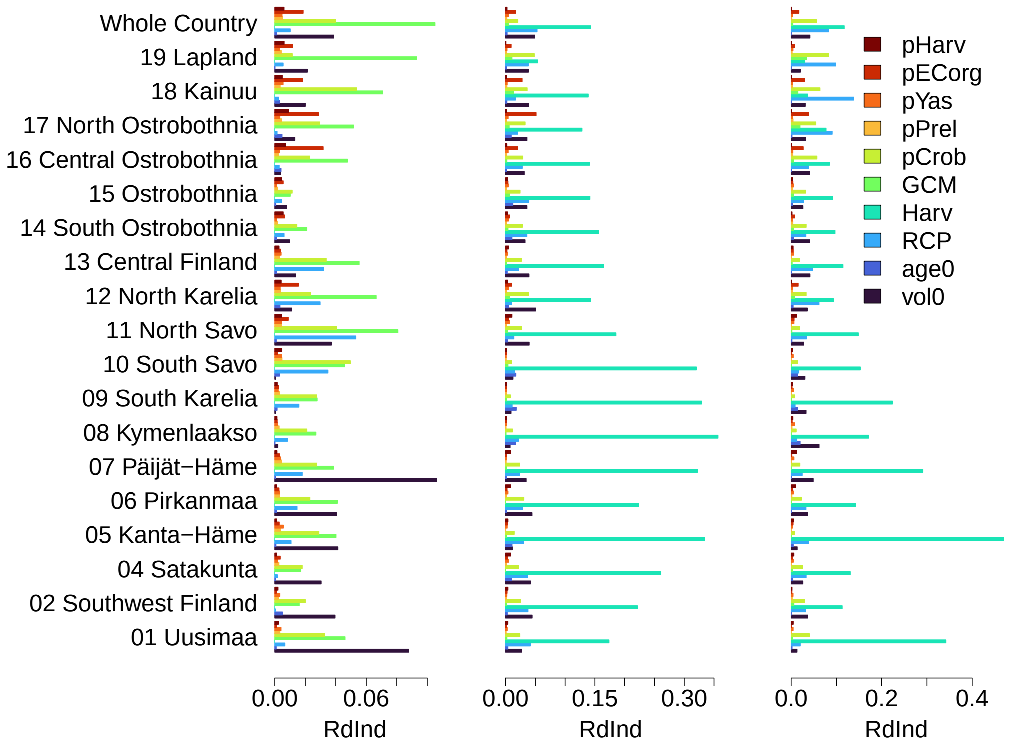

(a) 2015 **(b)** 2035 **(c)** 2050

Fig. S7. Redundancy index for different sources of uncertainty in NEE. For model parameter sets of CROBAS, PRELES, YASSO, organic soil emission factors and harvest level uncertainty, the highest parameter index value of the set specific Rdind is shown (pCrob, pPrel, pYas, pECorg and pHarv).

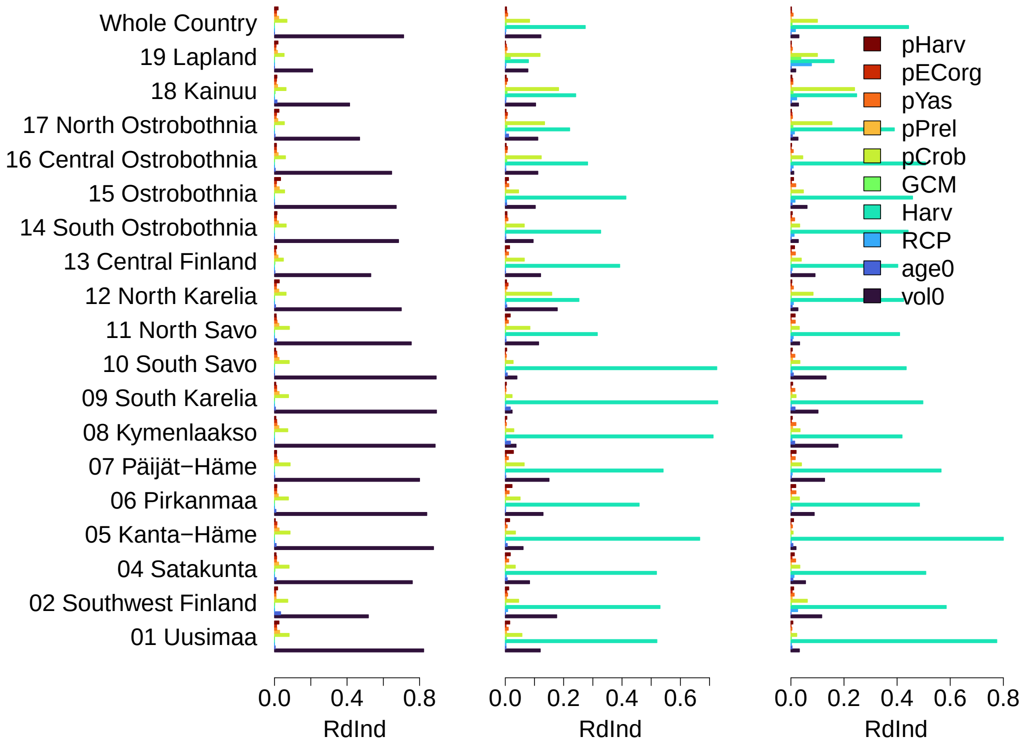

(a) 2015 **(b)** 2035 **(c)** 2050

Fig. S8. Redundancy index for different sources of uncertainty in tree biomass.

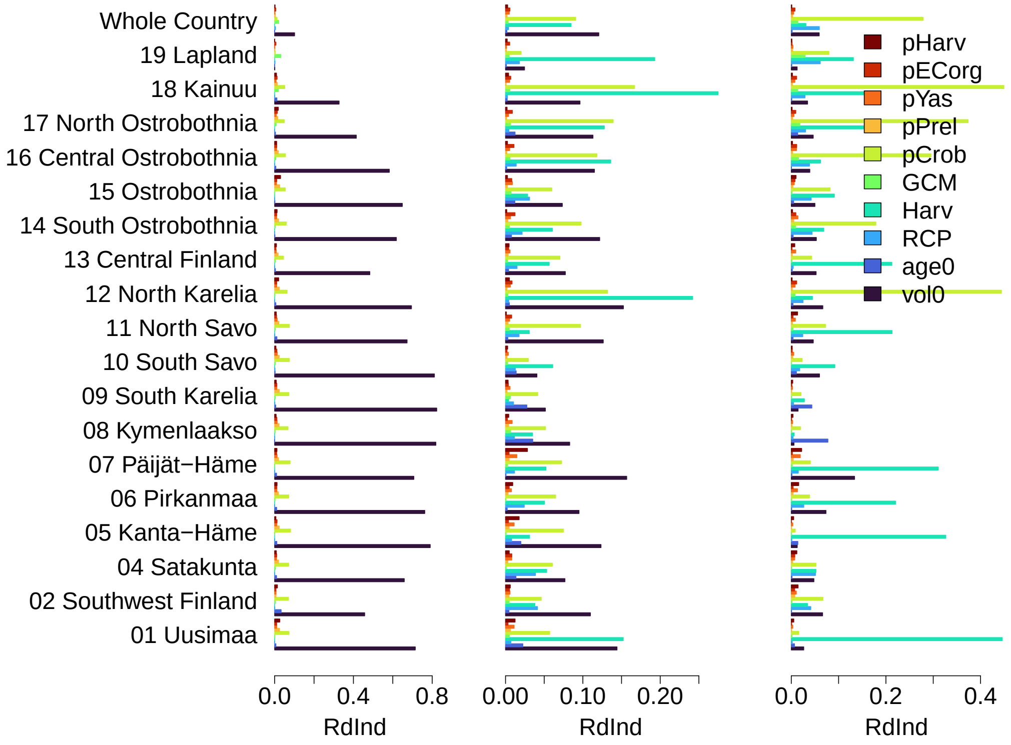

(a) 2015 **(b)** 2035 **(c)** 2050

Fig. S9. Redundancy index for different sources of uncertainty in ground vegetation biomass.

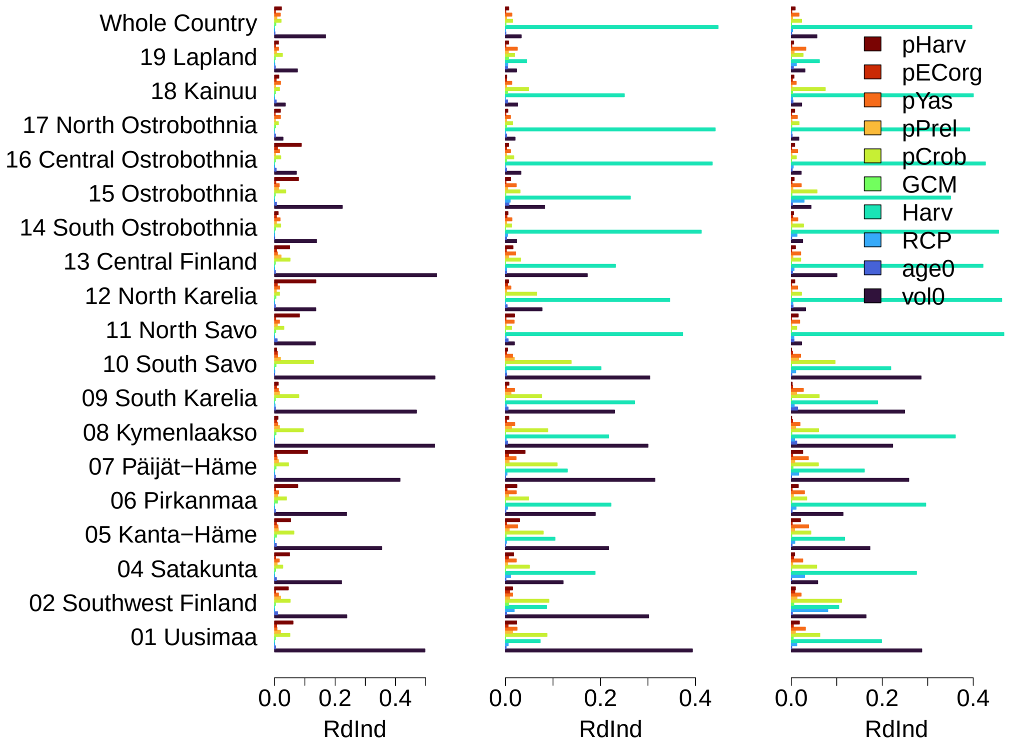

(a) 2015 **(b)** 2035 **(c)** 2050

Fig. S10. Redundancy index for different sources of uncertainty in soil carbon projections.
